# Microalgae as a Source of Photosensitizers: Analytical Strategies and Biomedical Use in Photodynamic Therapy

**DOI:** 10.3390/ph19010100

**Published:** 2026-01-06

**Authors:** Akzhol Kuanyshbay, Zhanar Iskakova, Yelaman Aibuldinov, Ainagul Kolpek, Yerbolat Tashenov, Nurgul Tursynova, Zhanar Tekebayeva, Zhanar Rakhymzhan, Aliya Temirbekova, Kamshat Kulzhanova, Bolatbek Zhantokov, Aidana Bazarkhankyzy

**Affiliations:** 1 Research Institute of New Chemical Technologies, L.N. Gumilyov Eurasian National University, Astana 010008, Kazakhstan; 2 Department of Chemistry, Faculty of Natural Sciences, L.N. Gumilyov Eurasian National University, Astana 010008, Kazakhstan; 3 Republican Collection of Microorganisms LPP, Astana 010000, Kazakhstan; 4 Department on Environmental Management and Engineering, Faculty of Natural Sciences, L.N. Gumilyov Eurasian National University, Astana 010008, Kazakhstan; zhanarr98@gmail.com (Z.R.);; 5 Department of General and Biological Chemistry, Faculty of Pharmacy, Astana Medical University, Astana 010000, Kazakhstan

**Keywords:** PDT, microalgae, *Chlorella* sp., photosensitizers, chlorophyll derivatives, carotenoids, subcellular localization, ROS, antimicrobial PDT

## Abstract

Photodynamic therapy (PDT) is an established light-based treatment modality that relies on the activation of photosensitizers to generate reactive oxygen species (ROS) and induce localized cytotoxicity. In recent years, microalgae have emerged as a promising and sustainable source of natural photosensitizers due to their ability to biosynthesize structurally diverse pigments with strong light-harvesting capacity. This review provides a comprehensive, application-oriented analysis of microalgae-derived photosensitizers, focusing on chlorophylls and their derivatives, carotenoids, and phycobiliproteins. Particular attention is given to analytical strategies for pigment extraction, purification, and characterization, as well as to photophysical properties, subcellular localization, and ROS-mediated mechanisms underlying photodynamic activity. Recent advances in the chemical modification of algal pigments, including chlorin-based derivatives and 5-aminolevulinic acid–related systems, are critically discussed in relation to structure–activity relationships and translational performance. The accumulated evidence demonstrates that microalgae-derived pigments and their synthetic analogues can achieve efficient singlet oxygen generation, organelle-specific phototoxicity, and favorable therapeutic selectivity. Taken together, these findings highlight microalgae as a renewable and versatile platform for developing next-generation photosensitizers with broad biomedical potential in oncology, dermatology, and antimicrobial photodynamic therapy.

## 1. Introduction

Photodynamic therapy (PDT) is a clinically established, minimally invasive treatment modality that combines a photosensitizer, light of an appropriate wavelength, and molecular oxygen to induce localized cytotoxicity. After light activation, the photosensitizer generates reactive oxygen species (ROS), which trigger oxidative damage and apoptosis in malignant or infected cells [1]. Photodynamic therapy (PDT) is widely used for dermatological and skin-cancer conditions such as actinic keratosis and basal cell carcinoma [2,3,4]. In addition, PDT has proven effective for early-stage esophageal cancer [5]. More recently, a growing number of clinical and preclinical studies have expanded the scope of PDT to include various solid tumors and ongoing cancer trials [6,7].

Despite its clinical potential, the therapeutic success of PDT is constrained by the physicochemical limitations of existing photosensitizers, such as poor water solubility, suboptimal tumor selectivity, slow clearance, and phototoxic side effects [8]. Moreover, many synthetic or semisynthetic agents require complex multistep synthesis and lack environmental sustainability. These limitations have motivated a search for natural, biocompatible, and renewable sources of photosensitizers with improved photophysical and biological profiles.

Microalgae represent a diverse group of photosynthetic microorganisms that naturally produce pigments with strong light-harvesting capacity, including chlorophylls, carotenoids, and phycobiliproteins. These molecules possess photophysical characteristics like clinically used porphyrin-type photosensitizers, making microalgae a promising biological platform for PDT research [9]. Species such as *Chlorella*, *Spirulina* (*Arthrospira*), *Haematococcus pluvialis*, *Dunaliella salina*, and *Phaeodactylum tricornutum* have been shown to accumulate pigments with high singlet oxygen yields and selective cytotoxicity under illumination [10,11,12].

Recent experimental studies have confirmed the photodynamic potential of purified algal pigments. Chlorophyll derivatives and pheophorbides extracted from *Chlorella* and marine diatoms induce mitochondria-dependent apoptosis in cancer cells after light activation [13,14]. Likewise, astaxanthin from *Haematococcus pluvialis* and fucoxanthin from *Phaeodactylum tricornutum* exhibit selective phototoxicity and modulate oxidative stress pathways [11,15]. C-phycocyanin from *Spirulina* has also demonstrated strong PDT-enhanced apoptotic activity in tumor models in vitro and in vivo [16].

The translational value of microalgae-derived pigments is supported by emerging preclinical and clinical evidence. A recent randomized controlled trial reported that a chlorin e6 derivative produced from *Spirulina* achieved significant clinical improvement in patients with moderate-to-severe rosacea, demonstrating both efficacy and favorable safety outcomes [17]. These findings suggest that algal pigments and their derivatives may serve as viable candidates for next-generation PDT agents in oncology, dermatology, and antimicrobial therapy.

This review provides an integrated analysis of microalgae as sources of natural photosensitizers, with emphasis on pigment diversity, extraction and purification strategies, photophysical properties, biological mechanisms of action, and biomedical applications. By summarizing recent advances in both natural and synthetic algal derivatives, we highlight the potential of microalgae-derived photosensitizers as sustainable and clinically relevant alternatives to conventional PDT agents. Despite extensive research on synthetic and semisynthetic photosensitizers, natural pigment-based systems, particularly those derived from microalgae, remain insufficiently systematized in the context of photodynamic therapy. Comparative insights into pigment classes, photophysical behavior, and mechanistic contributions to ROS generation are often fragmented across disciplines, highlighting the need for an integrated and application-oriented review.

## 2. Microalgal Pigments as Photosensitizers

**Figure 1:**
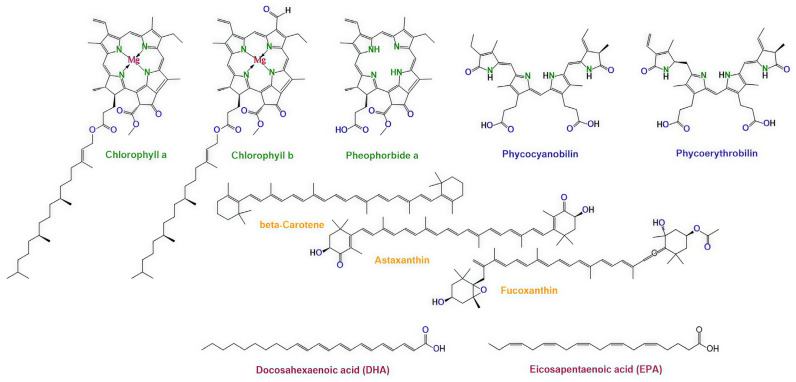
Representative chemical structures of microalgal pigments relevant to photodynamic therapy: chlorophyll a/b, pheophorbide a; carotenoids (β-carotene, astaxanthin, and fucoxanthin); phycocyanobilin (the chromophore of C-phycocyanin) and phycoerythrobilin (the chromophore of C-phycoerythrin); docosahexaenoic acid (DHA) and eicosapentaenoic acid (EPA) as a representative polyunsaturated fatty acid. Color coding is used to distinguish different classes of compounds: green—chlorophyll derivatives, orange—carotenoids, blue—phycobilin chromophores, and dark red—polyunsaturated fatty acids.

Microalgae produce a wide range of pigments that participate in photosynthesis, photoprotection, and intracellular energy transfer. Due to their extended conjugated systems and strong absorption in the visible region, many of these molecules exhibit photophysical properties like those of established photosensitizers. These include efficient light absorption, intersystem crossing to the triplet state, and the ability to generate singlet oxygen (^1^O_2_), which is the principal cytotoxic species in photodynamic therapy. The main pigment classes relevant to PDT are chlorophylls and their derivatives, carotenoids, and phycobiliproteins (Figure 1).

### 2.1. Chlorophylls and Chlorophyll Derivatives

Chlorophylls are porphyrin-type tetrapyrrolic pigments abundantly synthesized by green microalgae such as *Chlorella*, *Dunaliella*, and various diatoms [18]. Their absorption bands in the Soret (∼400–450 nm) and Q_y_ (∼660–680 nm) regions overlap with clinically relevant PDT wavelengths [19]. Chlorophylls readily undergo demetallation and de-esterification during extraction, yielding derivatives such as pheophytins and pheophorbides. These compounds often display improved photostability and higher quantum yields of singlet oxygen compared to their parent molecules [20].

Pheophorbide A extracted from green algae or diatoms has been shown to induce strong phototoxicity in tumor cells through ROS-mediated apoptosis, positioning it among the most extensively investigated natural chlorophyll-derived photosensitizers [14]. The intrinsic triplet-state reactivity of these derivatives makes them promising candidates for PDT applications in oncology and antimicrobial therapy.

### 2.2. Carotenoids

Carotenoids represent another major class of microalgal pigments. Species such as *Haematococcus pluvialis*, *Dunaliella salina*, and *Phaeodactylum tricornutum* accumulate astaxanthin, β-carotene, and fucoxanthin, respectively, often at high intracellular concentrations [21]. Although carotenoids are traditionally viewed as antioxidants, several studies have demonstrated that certain carotenoids can act as pro-oxidant agents under photoexcitation or in the presence of specific redox conditions. Fucoxanthin from diatoms displays strong absorption in the blue-green region and undergoes excited-state reactions that contribute to ROS formation [22]. Astaxanthin, while primarily quenching singlet oxygen, can also modulate oxidative pathways during illumination, influencing cellular redox balance [23]. Because carotenoids interfere with excited-state energy transfer, their role in PDT can be dual: they may act either as modulators of photodynamic efficiency or as auxiliary photosensitizers under specific conditions.

### 2.3. Phycobiliproteins

Cyanobacteria such as *Arthrospira (Spirulina)* produce phycobiliproteins, including C-phycocyanin (C-PC) and phycoerythrin (PE), which possess intense absorption bands in regions not covered by chlorophylls (∼550–650 nm). These biliproteins contain open-chain tetrapyrrole chromophores covalently bound to apoproteins and display strong fluorescence and efficient energy transfer [24].

C-phycocyanin has been shown to generate ROS under irradiation and to induce selective apoptosis in cancer cells via mitochondrial pathways [25]. Due to their photophysical properties, phycobiliproteins are increasingly recognized as potential natural photosensitizers and fluorescent probes for combined imaging–therapy strategies.

### 2.4. Lipids and Polyunsaturated Fatty Acids (PUFAs)

Some microalgae, particularly marine species, produce high levels of polyunsaturated fatty acids (PUFAs) and other photoactive lipids. Although these compounds are not classical photosensitizers, they can undergo photooxidation and enhance lipid peroxidation during PDT, contributing to membrane destabilization and amplifying phototoxic responses [26]. Their presence may therefore modulate the overall photodynamic effect of pigment mixtures extracted from algal biomass.

A summary of representative microalgal species, their dominant pigments, and their reported photophysical or photodynamic properties is presented in Table 1.

The photodynamic efficacy of microalgae-derived pigments is governed by their intrinsic photophysical properties and their ability to generate reactive oxygen species upon light activation. Depending on pigment structure, cellular environment, and oxygen availability, different photochemical pathways may contribute to the overall biological response. A detailed discussion of these photochemical mechanisms and their biological implications is provided in Section 3.

## 3. Mechanisms of Photodynamic Action of Microalgae-Derived Photosensitizers

The photodynamic activity of microalgae-derived photosensitizers is primarily determined by their photophysical behavior upon light activation, subcellular localization, and the nature of reactive oxygen species (ROS) generated during irradiation. Unlike conventional synthetic photosensitizers, algal pigments represent structurally diverse natural systems that may engage multiple photochemical and biological pathways, resulting in selective cytotoxicity toward malignant or microbial cells.

### 3.1. Type I and Type II Photochemical Pathways

**Figure 2:**
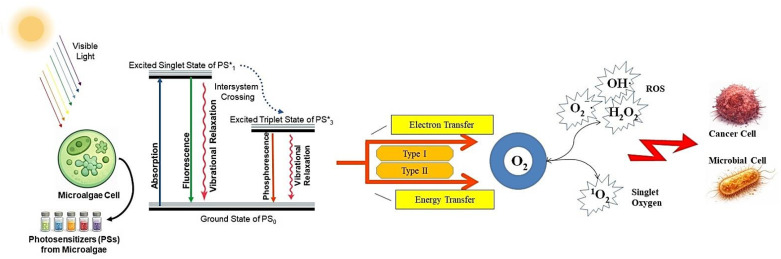
Schematic representation of the photodynamic mechanism of action of microalgae-derived photosensitizers. Upon absorption of visible light, the photosensitizer (PS) is excited from the ground state to the excited singlet state, followed by intersystem crossing to the excited triplet state. From this state, photochemical reactions proceed via energy transfer to molecular oxygen, generating singlet oxygen (^1^O_2_) (Type II pathway), or via electron transfer reactions leading to the formation of other reactive oxygen species (Type I pathway). The generated reactive oxygen species induce oxidative damage in cancer or microbial cells, resulting in photodynamic cytotoxicity.

Upon absorption of light at appropriate wavelengths, microalgae-derived photosensitizers are promoted from the ground singlet state to an excited singlet state, followed by intersystem crossing to a long-lived triplet state. This excited triplet state can participate in two principal photodynamic pathways: Type I and Type II mechanisms (Figure 2). In the Type II pathway, energy transfer from the triplet photosensitizer to molecular oxygen results in the formation of singlet oxygen (^1^O_2_), which is widely recognized as the dominant cytotoxic species in photodynamic therapy [1]. Chlorophyll derivatives and phycobiliproteins, owing to their efficient light absorption and favorable triplet-state lifetimes, predominantly operate via this mechanism and exhibit high singlet oxygen yields [35,36,37].

In parallel, certain microalgae-derived pigments may also engage in Type I photochemical reactions, which involve electron or hydrogen transfer between the excited photosensitizer and surrounding biomolecules. These reactions generate radical species such as superoxide anions, hydrogen peroxide, and hydroxyl radicals [38,39]. Carotenoids and lipid-associated pigments, particularly under low-oxygen or hypoxic conditions, are more likely to contribute to Type I processes, thereby expanding the photodynamic response beyond singlet oxygen generation [40,41,42].

Importantly, the balance between Type I and Type II mechanisms depends not only on the chemical structure of the pigment but also on local oxygen availability, light dose, and cellular microenvironment. This dual photochemical behavior provides microalgae-derived photosensitizers with functional flexibility and may enhance PDT efficacy in heterogeneous biological tissues.

### 3.2. Subcellular Localization and Organelle-Specific Phototoxicity

A key determinant of photodynamic selectivity is the subcellular localization of photosensitizers prior to light activation. Numerous studies have demonstrated that pigments derived from microalgae preferentially accumulate in organelles that are highly sensitive to oxidative stress, including mitochondria, lysosomes, and the endoplasmic reticulum. A summary of representative microalgal pigments, their dominant subcellular localization, and associated photodynamic effects is provided in Table 2.

Chlorophyll derivatives and pheophorbides obtained from species such as *Chlorella* and marine diatoms have been shown to localize predominantly in mitochondria and lysosomes. Upon photoactivation, localized ROS generation disrupts mitochondrial membrane integrity, leading to loss of membrane potential and activation of intrinsic apoptotic signaling. Similarly, lysosomal photodamage results in membrane permeabilization and release of hydrolytic enzymes, which further amplify cell death pathways [8,14,43].

Carotenoids such as fucoxanthin and astaxanthin also demonstrate selective intracellular distribution, frequently associated with mitochondrial membranes and lipid-rich compartments. Their localization enables effective ROS-mediated lipid peroxidation, which contributes to membrane destabilization and enhances phototoxic outcomes [31,42,44,45,46].

Phycobiliproteins, particularly C-phycocyanin from *Arthrospira* (*Spirulina*), exhibit distinct intracellular behavior and have been reported to accumulate in lysosomal or cytoplasmic regions. Upon irradiation, these pigments induce ROS-dependent apoptosis while simultaneously modulating cellular stress responses, highlighting their dual cytotoxic and regulatory roles [25,33,47,48,49].

### 3.3. ROS-Mediated Cell Death and Microenvironment Modulation

Regardless of the dominant photochemical pathway, ROS generation represents the central mediator of PDT-induced cytotoxicity. Reactive oxygen species produced by microalgae-derived photosensitizers oxidize lipids, proteins, and nucleic acids, ultimately leading to programmed cell death through apoptosis or, in some cases, regulated necrosis [8,39].

Beyond direct intracellular damage, accumulating evidence suggests that photodynamic action also influences the surrounding cellular microenvironment. ROS-mediated stress can disrupt tumor vasculature, enhance membrane permeability, and promote the release of damage-associated molecular patterns (DAMPs), which collectively contribute to improved therapeutic outcomes [6,8].

Additionally, lipid-rich extracts and polyunsaturated fatty acids present in certain microalgal species may undergo photooxidation, generating cytotoxic lipid peroxides that further amplify photodynamic efficacy. Such effects are particularly relevant in antimicrobial PDT, where membrane damage plays a dominant role in microbial inactivation [26,42].

Together, these observations underscore the multifaceted nature of microalgae-derived photosensitizers, which function not only as light-activated cytotoxic agents but also as modulators of cellular and microenvironmental responses.

## 4. Extraction and Purification of Microalgae-Derived Photosensitizers

The efficient extraction and purification of photosensitizing compounds from microalgae are critical steps that directly influence their photophysical properties, biological activity, and suitability for biomedical applications. Unlike synthetic photosensitizers, algal pigments are embedded within complex cellular matrices and are often co-extracted with proteins, lipids, and polysaccharides, necessitating tailored analytical strategies to achieve adequate purity and functional integrity.

### 4.1. Pretreatment and Cell Disruption Strategies

Microalgal cell walls exhibit considerable structural diversity, ranging from relatively fragile membranes in *Dunaliella* species to highly resilient polysaccharide- or silica-containing walls in *Chlorella* and diatoms. As a result, effective cell disruption is a prerequisite for pigment recovery. Common pretreatment approaches include mechanical methods (bead milling, ultrasonication, high-pressure homogenization) and non-mechanical techniques such as enzymatic digestion or osmotic shock [50,51,52,53].

Mechanical disruption methods are widely applied due to their scalability and compatibility with downstream extraction processes. However, excessive shear forces may promote pigment degradation or alter excited-state behavior, particularly for chlorophyll derivatives and phycobiliproteins. Consequently, extraction protocols must balance disruption efficiency with preservation of photodynamic functionality.

### 4.2. Solvent-Based Extraction of Pigment Classes

The choice of extraction solvent is strongly dependent on pigment polarity and molecular structure. Chlorophylls and their derivatives are typically extracted using organic solvents such as acetone, ethanol, methanol, or their aqueous mixtures. Among these, ethanol has gained increasing attention as a green solvent compatible with pharmaceutical applications and regulatory requirements [54,55,56].

Carotenoids, including β-carotene, astaxanthin, and fucoxanthin, require nonpolar or moderately polar solvents (e.g., hexane, ethyl acetate, acetone) to ensure efficient recovery. Supercritical CO_2_ extraction has emerged as a promising alternative for carotenoid isolation, offering high selectivity, solvent-free extracts, and reduced thermal degradation [56,57,58].

In contrast, phycobiliproteins such as C-phycocyanin are water-soluble and are commonly extracted using buffered aqueous systems. Mild extraction conditions are essential to preserve protein conformation and fluorescence properties, which are closely linked to their photodynamic and imaging performance [59,60].

### 4.3. Purification and Fractionation Techniques

Crude algal extracts often contain complex mixtures of pigments and biomolecules that may interfere with PDT performance or reproducibility. Chromatographic purification is therefore essential to isolate individual photosensitizers and to enable reliable structure–activity correlations.

Liquid–liquid partitioning is frequently used as a preliminary fractionation step to separate pigments based on polarity. This is typically followed by column chromatography using silica gel or polymeric stationary phases. For higher purity requirements, high-performance liquid chromatography (HPLC) and preparative HPLC are widely employed, particularly for chlorophyll derivatives and carotenoids [61,62]. Advanced techniques such as high-performance countercurrent chromatography (HPCCC) and Elution-Extrusion Countercurrent Chromatography (EECCC) have been successfully applied to the isolation of fucoxanthin and other labile pigments, offering high recovery yields without irreversible adsorption to solid supports. These approaches are particularly valuable for preserving photophysical properties relevant to PDT [63,64].

### 4.4. Analytical Characterization and Quality Control

Following purification, comprehensive analytical characterization is required to confirm pigment identity, purity, and photodynamic suitability. UV–Vis spectroscopy is routinely used to assess absorption maxima and molar extinction coefficients, while fluorescence spectroscopy provides insight into excited-state behavior. Chromatographic purity is typically evaluated by HPLC, often coupled with diode-array detection or mass spectrometry.

Structural confirmation is achieved using spectroscopic techniques such as FT-IR and NMR, whereas singlet oxygen generation and ROS production are commonly assessed using chemical probes and photochemical assays. These analytical steps are essential for correlating extraction and purification conditions with biological performance in PDT [65,66].

Despite significant progress, several challenges remain in the extraction and purification of microalgae-derived photosensitizers. These include batch-to-batch variability in biomass composition, pigment instability during processing, and scalability constraints for pharmaceutical translation. Integrating green extraction technologies with advanced chromatographic purification and standardized analytical workflows represents a promising strategy to overcome these limitations.

In summary, continued optimization of extraction and purification methodologies will be crucial for unlocking the full potential of microalgae as a sustainable and versatile source of photosensitizers for photodynamic therapy.

## 5. Synthetic Derivatives of Microalgae-Derived Photosensitizers and Their Biomedical Applications

Natural pigments isolated from microalgae, particularly chlorophylls and their degradation products, provide versatile molecular scaffolds for the development of synthetic photosensitizers with enhanced photodynamic performance. While native algal pigments already exhibit notable phototoxicity upon light activation, chemical modification enables fine-tuning of their absorption properties, photostability, subcellular targeting, and reactive oxygen species (ROS) generation, thereby improving their translational potential for clinical photodynamic therapy (PDT).

### 5.1. Chlorin-Based Derivatives Originating from Microalgal Chlorophylls

Among microalgae-derived pigments, chlorophyll *a* and its catabolites (chlorin e6 and pheophorbide A) represent the most extensively explored precursors for synthetic photosensitizer development. These tetrapyrrolic structures possess strong absorption in the red and near-infrared (NIR) regions, which is advantageous for deeper tissue penetration during PDT.

**Figure 3:**
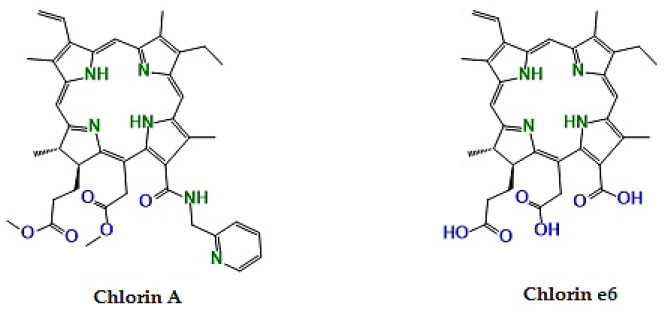
General chemical structures of chlorophyll-derived chlorins, including chlorin A and chlorin e6, are commonly used as scaffolds for the development of synthetic photosensitizers due to their strong absorption in the red spectral region and high singlet oxygen generation efficiency.

One representative example is Chlorin A (Figure 3), a pyridyl-substituted chlorin derivative synthesized from chlorophyll-based precursors isolated from *Chlorella* and *Spirulina*. This compound exhibits red-shifted absorption (≈664–667 nm), high singlet oxygen yield, and pronounced phototoxicity in vivo, resulting in significant tumor regression in murine cancer models. These properties highlight how modest structural modification of natural chlorophyll frameworks can substantially enhance PDT efficacy [67,68]. Similarly, chlorin e6 (naturally occurring in microalgae as a chlorophyll *a* derivative) has served as a central platform for numerous synthetic analogues aimed at improving tumor selectivity and photophysical performance [68,69,70].

### 5.2. Functional Modifications Enhancing Photodynamic Performance

Targeted functionalization of chlorin e6 has proven particularly effective in overcoming limitations associated with conventional photosensitizers, such as insufficient cellular uptake, photobleaching, and reduced efficacy under hypoxic conditions. A notable strategy involves the introduction of guanidinium or other cationic moieties, which enhance electrostatic interactions with negatively charged cellular membranes and promote organelle-specific accumulation.

**Figure 4:**
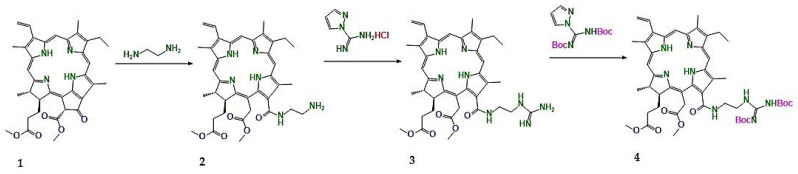
Schematic illustration of representative functional modifications of chlorin e6, including guanidine conjugation and side-chain derivatization, leading to enhanced photostability, increased reactive oxygen species generation, and improved photocytotoxicity of chlorin-based photosensitizers. (**1**) Chlorin e6. (**2**) Chlorin e6 modified via aliphatic diamine side-chain conjugation. (**3**) Guanidine-functionalized chlorin e6 derivative. (**4**) Boc-protected guanidine-functionalized chlorin e6 derivative.

For example, guanidine-conjugated chlorin e6 derivatives (3–4) have demonstrated superior photostability, enhanced ROS generation, and increased photocytotoxicity compared with clinically approved photosensitizers such as m-THPC (Figure 4). Fluorescence microscopy studies revealed preferential localization of these derivatives in mitochondria, lysosomes, and the endoplasmic reticulum (organelles that are highly susceptible to ROS-mediated damage), resulting in robust apoptosis induction in lung and gastrointestinal cancer models [71]. Importantly, these derivatives retain low dark toxicity while exhibiting strong light-dependent cytotoxicity, a key requirement for safe clinical translation.

### 5.3. ALA-Based Photosensitizer Derivatives

In addition to chlorin-based compounds, synthetic derivatives of 5-aminolevulinic acid (5-ALA) represent another important class of photosensitizers linked to microalgal metabolism. Although 5-ALA is a universal biosynthetic precursor in the tetrapyrrole pathway and has been reported in some microalgal systems, its synthetic ester derivatives have been engineered to improve bioavailability and membrane permeability [72].

Alkylated ALA derivatives (e.g., ALA-methyl and ALA-hexyl esters) exhibit enhanced intracellular conversion to protoporphyrin IX (PpIX), leading to efficient ROS production upon light activation. These compounds have demonstrated rapid bacterial eradication in antimicrobial photodynamic therapy (aPDT) models, as well as promising results in wound healing and localized infection control [72,73,74,75].

The dual applicability of ALA-based derivatives in both oncology and antimicrobial PDT underscores the versatility of tetrapyrrole-based photosensitizers derived from or inspired by microalgal metabolic pathways.

### 5.4. Translational Perspectives and Structure–Activity Relationships

Collectively, studies on synthetic derivatives of microalgae-derived photosensitizers reveal clear structure–activity relationships. Red-shifted absorption, cationic functional groups, and organelle-targeting motifs consistently correlate with enhanced photodynamic efficacy, improved tumor selectivity, and broader therapeutic windows (Table 3).

These synthetic agents exemplify a translational continuum, in which naturally occurring algal pigments serve as renewable chemical templates that can be systematically optimized for biomedical use. Importantly, several chlorin-based derivatives and ALA formulations have progressed to preclinical and early clinical evaluation, demonstrating the feasibility of translating microalgae-inspired photosensitizers into practical PDT applications [76,77].

## 6. Conclusions

Microalgae represent a biologically rich and chemically versatile source of photosensitizers with significant relevance for photodynamic therapy. The diversity of algal pigments—ranging from chlorophyll derivatives and carotenoids to phycobiliproteins and photoactive lipids—provides a unique foundation for exploring natural photodynamic systems that combine efficient light absorption with ROS-mediated biological activity. As demonstrated throughout this review, many microalgae-derived pigments exhibit photophysical characteristics comparable to clinically used photosensitizers, including favorable absorption profiles, effective singlet oxygen generation, and selective intracellular localization.

Beyond their intrinsic photodynamic properties, advances in extraction, purification, and analytical characterization have enabled the reliable isolation and functional assessment of algal photosensitizers, supporting meaningful structure–activity correlations. Furthermore, the chemical modification of naturally occurring pigments, particularly chlorophyll-based scaffolds, has led to synthetic derivatives with improved photostability, enhanced cellular uptake, and increased therapeutic selectivity. These developments underscore the translational potential of microalgae-inspired photosensitizers and demonstrate how natural molecular frameworks can be systematically optimized for biomedical use.

Importantly, microalgae-derived systems also offer broader advantages related to sustainability, scalability, and biocompatibility, aligning well with current demands for environmentally responsible pharmaceutical development. While challenges remain—including biomass variability, pigment stability, and clinical standardization—the growing body of preclinical and early clinical evidence supports continued investigation into algal photosensitizers as viable alternatives to conventional synthetic agents.

In conclusion, the integration of microalgal biotechnology, analytical chemistry, and photodynamic medicine opens new avenues for the rational design of next-generation photosensitizers. Continued interdisciplinary efforts are expected to further expand the clinical applicability of microalgae-derived pigments, reinforcing their role as a renewable and innovative resource in the future landscape of photodynamic therapy.

## Figures and Tables

**Table 1:** Representative microalgal species, major pigment classes, and documented photophysical/photodynamic properties.

Species	Pigment(s)	Peak Absorption	Reported Photodynamic/Photophysical Properties	Refs.
*Chlorella vulgaris*	Chlorophyll a/b, pheophorbide a	430, 665 nm	ROS generation, phototoxicity in vitro	[14,20]
*Haematococcus pluvialis*	Astaxanthin	480–510 nm	Light-dependent redox modulation	[27,28]
*Dunaliella salina*	β-carotene	450–500 nm	Pro-oxidant activity under irradiation	[29,30]
*Phaeodactylum tricornutum*	Fucoxanthin	450–560 nm	ROS involvement, anticancer potential	[31,32]
*Arthrospira platensis* (Spirulina)	C-phycocyanin	615–640 nm	PDT-induced apoptosis; ROS generation	[25,33,34]

**Table 2:** Representative microalgal pigments, dominant subcellular localization, and associated photodynamic effects.

Microalgal Source/Pigment	Major Pigment Class	Dominant Subcellular Localization	Main Photodynamic Outcome	Notes/Relevance for PDT	Refs.
*Chlorella* spp.	Chlorophyll a/b, pheophorbide a	Mitochondria, lysosomes	ROS-mediated apoptosis; mitochondrial dysfunction	High singlet oxygen yield; reference model for algal PDT	[14,20]
*Arthrospira* (*Spirulina*) *platensis*	C-phycocyanin	Lysosomes, cytoplasm	PDT-induced apoptosis; modulation of oxidative stress	Water-soluble pigment; dual cytotoxic and immunomodulatory effects	[25,33]
*Haematococcus pluvialis*	Astaxanthin	Mitochondrial membranes	Redox modulation; enhanced phototoxicity under irradiation	Acts as ROS modulator; improves PDT selectivity	[23,27]
*Dunaliella salina*	β-Carotene	Cellular membranes, cytoplasm	Pro-oxidant activity under photoexcitation; ROS balance	Effective under blue–green light; anticancer and antimicrobial PDT	[29,41]
*Phaeodactylum tricornutum*	Fucoxanthin	Mitochondria	ROS-mediated apoptosis; lipid peroxidation	Effective under blue–green light; anticancer and antimicrobial PDT	[31,32]
*Nannochloropsis* spp.	PUFAs (EPA, DHA)	Cellular membranes	Photooxidation-driven lipid peroxidation	Enhances membrane damage; relevant for antimicrobial PDT	[26,42]
*Porphyridium cruentum*	Phycoerythrin, sulfated polysaccharides	Cytoplasm, extracellular matrix	ROS-mediated cytotoxicity; immunomodulation	Supports wound healing and TME modulation	[12,24]

**Table 3:** Synthetic derivatives of microalgae-derived photosensitizers, structural modifications, and biomedical outcomes.

Synthetic Derivative	Natural Precursor	Structural Modification	Key Advantages	Reported Outcomes	Refs.
Chlorin A	Chlorin E6 (from chlorophyll A, *Chlorella*, *Spirulina*)	Pyridyl substitution	Longer λ absorption (664–667 nm), higher singlet oxygen yield	Strong in vivo antitumor activity (murine esophageal cancer), deep tissue penetration	[67,68]
Guanidine-conjugated chlorins (compounds **3**, **4**)	Chlorin E6 (*Chlorella*, *Spirulina*)	Guanidinium groups at C13	Improved cellular uptake, ROS under hypoxia, and enhanced stability	Outperformed m-THPC in A549 models; mitochondrial/lysosomal localization; strong apoptosis induction	[71]
ALA derivatives (ALA-methyl, ALA-hexyl)	5-ALA (biosynthetic precursor present in microalgae)	Alkyl substitution of ALA	Higher bioavailability, improved uptake, broad antimicrobial spectrum	Rapid bacterial eradication in vitro, wound healing acceleration in vivo, and low systemic toxicity	[72,75]
Chlorin E6 diaminoethylcarboxamide	Pheophorbide A	Ethylenediamine substitution	Higher tumor selectivity, enhanced mitochondrial localization	Strong apoptotic induction in cancer cells, precursor for further chlorin analogues	[36,65]

## Data Availability

No new data were created or analyzed in this study. Data sharing is not applicable to this article.
